# Supplementary material for: Stimulated emission of Cooper pairs in a high-temperature cuprate superconductor
Source: Sci Rep. 2016 Jul 1;6:29100. doi: 10.1038/srep29100 (PMC4929572; doi:10.1038/srep29100)
Supplement: Supplementary Information [file srep29100-s1.pdf]

SUPPLEMENTARY INFORMATION FOR

Stimulated emission of Cooper pairs in a high-temperature cuprate superconductor

W. T. Zhang, C. L. Smallwood, T. L. Miller, Y. Yoshida, H. Eisaki, R. A. Kaindl, D.-H. Lee, and A. Lanzara

Supplementary Figure 1

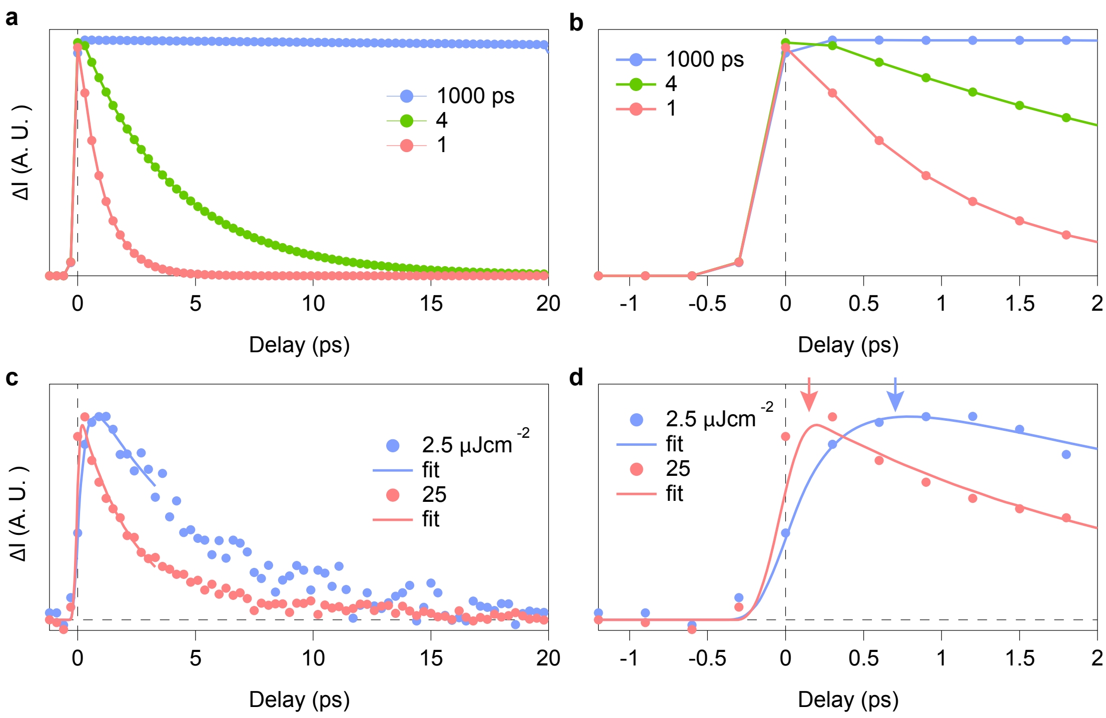

Supplementary Figure 1 | Comparison between simulated and experimental decay curves. a, Simulated decay curves with zero buildup time and decay time 1, 4, and 1000 ps, convolved with a Gaussian resolution function (FWHM = 300 fs). **c**, The decay curves at pump fluence 2.5 μJcm^{-2} (below F_c) and 25 μJcm^{-2} (above F_c) for an underdoped 78 K sample. Corresponding decay curves in **a** and **c** at delay time between -1.2 and 2 ps are shown in **b** and **d** respectively.

Supplementary Figure 2

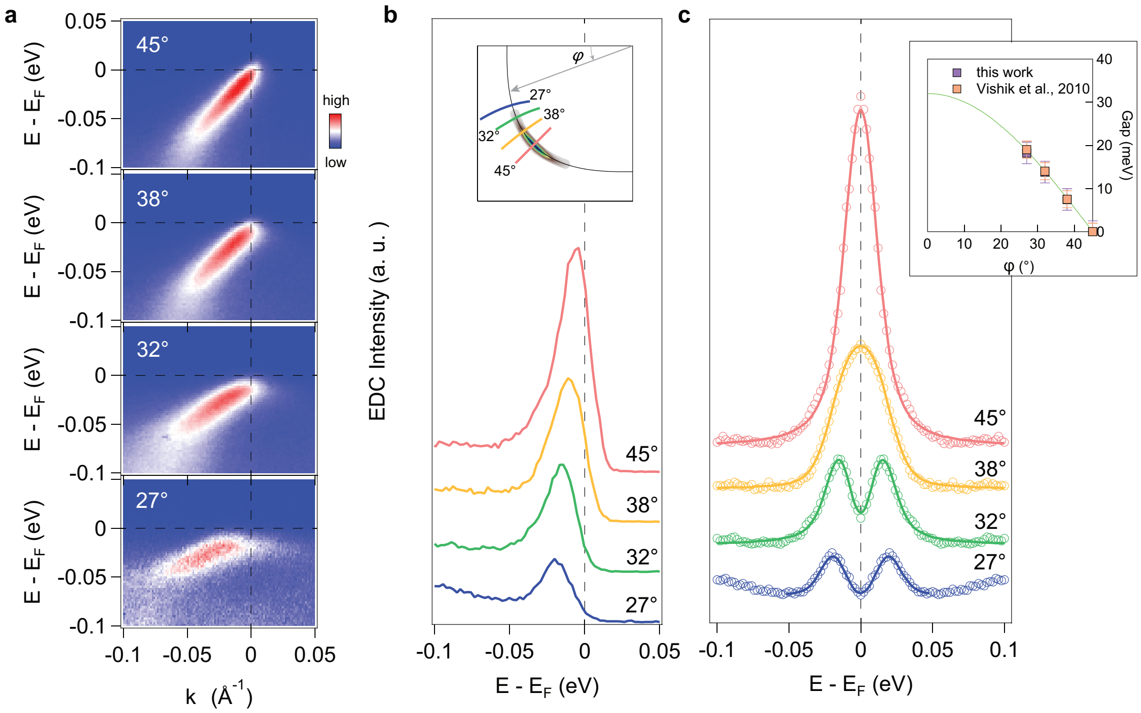

Supplementary Figure 2 | Extraction of energy gaps on the Fermi surface. a, Equilibrium photoemission spectra as a function of energy and momentum represented by false color. Cuts in the Brillouin zone are shown in inset of **b**. **b**, EDCs at the Fermi momentum for the corresponding cuts shown in the inset. **c**, Symmetrized energy distribution curves at each Fermi momentum and fittings using a phenomenological model. The inset in **c** is the energy gap as a function of Fermi surface angle extracted from the fitting. The energy gap in similar doping of samples is also shown for comparison (Vishik et al., Physical Review Letters 104, 207002 (2010)).

Supplementary Figure 3

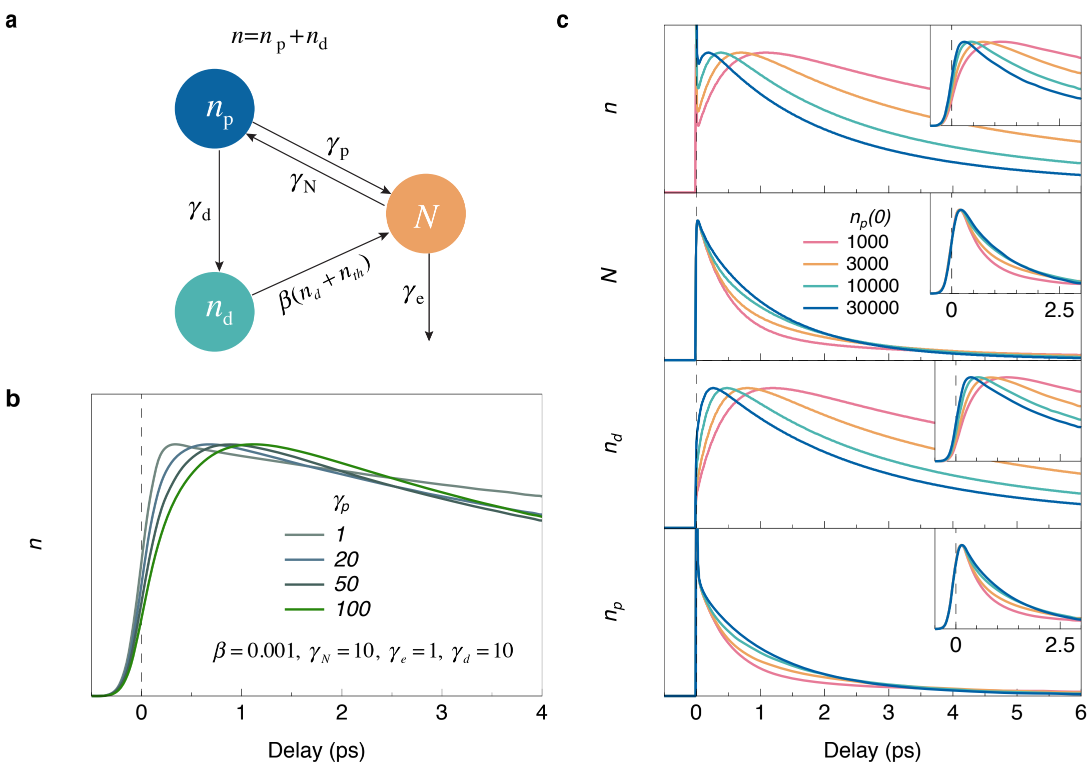

Supplementary Figure 3 | Numerical solution of *n*, *N*, n_d, and n_p. a, Schematization of the rate equations for the number of non-equilibrium electrons and bosons. **b**, Numerical solutions of the number of non-equilibrium electrons (n) at different stimulated pairing rate γ_p. Corresponding parameters are noted. All the curves are convolved with a system time resolution of 300 fs. **c**, Numerical solutions with $\gamma_p = 100$ for different $n_p(0)$. Insets are the corresponding numerical solutions convolved with time resolution. All the curves are normalized to the same height.

Supplementary Discussion 1

The observed delay of the initial build-up of non-equilibrium quasiparticles between low and high pump fluence cannot be attributed to a time-resolution effect, as demonstrated in the following simulation and discussion. Usually, limited time resolution would broaden the initial buildup of number of non-equilibrium quasiparticles, but here such broadening is too small to explain our data.

Simulation was done based on Eq. (1) in the main text by setting the buildup time $t_{buildup}$ = 0 ps. A Gaussian time-resolution function with FWHM = 300 fs was convolved in each simulated decay curves. Decay curves with decay time 1, 4, and 1000 ps are shown in Supplementary Figure 1a, which shows no apparent delay between fast and slow decay curves. Supplementary Figure 1c shows decay curves for pump fluences far below (2.5 μJcm^{-2}) and above (25 μJcm^{-2}) the critical fluence shown in Fig. 2a of the main text, and there is an apparent difference in buildup time.
A delay between slow decay curves and fast decay curves is more obvious in Supplementary Figure 1d, which was zoomed in between delay times -1.2 ps and 2 ps, but negligible delay in the simulated data in supplementary Figure 1b.

Supplementary Discussion 2

Energy gaps on the Fermi surface shown in Fig. 3**e** in the main text are from fitting the symmetrized EDCs at the Fermi momentum to phenomenological single quasiparticle spectral functions at the Fermi momentum (ref. 19).

Supplementary Figure 2**a** shows the equilibrium photoemission intensity as a function of energy and momentum, for the four cuts shown in the inset of supplementary Figure 2**b**. With energy resolution of 23 meV, the opening of the energy gap open from node to off-node can be identified in the photoemission image. Supplementary Figure 2**b** show the energy distribution curves (EDCs) at the corresponding Fermi momenta, and supplementary Figure 2**c** show the symmetrized EDCs centered at the Fermi level with an assumption of particle-hole symmetry that $A(k_F,\omega) = A(k_F,-\omega)$. Energy gaps are extracted by fitting the symmetrized EDCs to a phenomenological model for single particle spectra function in superconducting state (ref. 19).
The fitting function is

$$I(\omega) = C_0 + C_1|\omega| + \frac{C_2\Gamma}{(\omega - \Delta^2/\omega)^2 + \Gamma^2}$$

where Δ is the energy gap, Γ is the peak width, and $C_0 + C_1|\omega|$ is the incoherent background. Because of limited energy resolution of 23 meV of ultra short pulse, a Gaussian resolution function with FWHM = 23 meV is convolved with the above function during the fitting.

The energy gap as a function of the Fermi surface angle φ extracted in this way is shown in the inset of supplementary Figure 2**c**. The obtained energy gap is consistent with previous results from high-resolution photoemission.

Supplementary Discussion 3

Since the recombination of non-equilibrium electrons is related to the underlying condensate, we define two kinds of non-equilibrium electrons in the system: a), n_p, the number of "paired" non-equilibrium electrons, whose time reversal partners are also excited b) n_d, number of "unpaired" non-equilibrium electrons, whose time reversal partners are not simultaneously excited

To describe the dynamics of a photo-excited system and as schematized in (supplementary Figure 3**a**), we propose augmented Rothwarf-Taylor equations

$$\dot{n} = -\beta n_{\mathrm{d}}^2 + 2\gamma_{\mathrm{N}} N - 2\beta n_{\mathrm{d}} n_{\mathrm{th}} - \gamma_{\mathrm{p}} n_{\mathrm{p}} \quad \text{(S16)}$$

$$\dot{N} = \beta n_{\mathrm{d}}^2/2 - \gamma_{\mathrm{N}} N + \beta n_{\mathrm{d}} n_{\mathrm{th}} - \gamma_{\mathrm{e}} N + \gamma_{\mathrm{p}} n_{\mathrm{p}}/2 \quad \text{(S17)}$$

$$\dot{n}_{\mathrm{p}} = -\gamma_{\mathrm{d}} n_{\mathrm{p}} + 2\gamma_{\mathrm{N}} N - \gamma_{\mathrm{p}} n_{\mathrm{p}} \quad \text{(S18)}$$

$$n = n_{\mathrm{d}} + n_{\mathrm{p}} \quad \text{(S19)}$$

where n is the number of excited electrons, N is the number of excited bosons at the two gap energy, n_{th} is the number of thermal populated electrons, β is the recombination coefficient, γ_N is the pair breaking coefficient by bosons, γ_e is the rate at which two-gap energy phonons are removed from the interacting system, γ_d is the rate at which "paired" non-equilibrium electrons scatter and become "unpaired" , and γ_p is the rate of stimulated emission into the existing superconducting condensate. The stimulated emission factor is also encoded in β, which is proportional to the number of condensed Cooper pairs in the system. Originally, the Rothwarf-Taylor model was derived to describe the dynamics of Cooper pair formation by injecting normal electrons to a condensed pairing system (ref. 16), in which the normal electrons are all“un-paired” excited quasiparticles, unlike the photon-induced non-equilibrium electrons here.

Supplementary Figure 3b shows the numerical solutions of n for different γ_p at zero temperature ($n_{th} = 0$). Considering that the Fermi velocity in Bi2212 is about 3×10^5 ms^{-1}, the lifetime of quasiparticles is about 200 fs, the farthest distance of one quasiparticle moving in 6 ps of interest is about 300 nm with a random walk model, corresponding about 40000 pairs in this region (coherence length is about 1.5 nm). Thus we set $n_p(0) = 1000$, which corresponds a perturbation to the system by pump pulse, as the initial number of non-equilibrium electrons at the pairing positions. For small γ_p, solutions of the rate equations give faster buildup time in n, suggesting that to get such a long buildup time as the one shown in Fig. 2 of the main text, the pair formation from stimulated emission must be very fast. To simulate a buildup time of 1 ps, γ_p must be around 100 ps^{-1}, suggesting that the time of the stimulated emission in Bi2212 is on the order of or shorter than 10 fs. Note that these simulations use γ_e is on the order of 1 ps^{-1}, which shows that stimulated emission may cause a longer buildup time even when the system is in the weak bottleneck regime. Moreover, simulations indicate that for higher excitation density, the build-up of non-equilibrium electrons is faster (supplementary Figure 3c), consistent with the data in Fig. 2 in the main text.
